# An Update on S100A16 in Human Cancer

**DOI:** 10.3390/biom13071070

**Published:** 2023-07-03

**Authors:** Suyog Basnet, Evan Michael Vallenari, Urusha Maharjan, Sunita Sharma, Olaf Schreurs, Dipak Sapkota

**Affiliations:** 1 Curida Diatec, 1364 Oslo, Norway; 2 Department of Oral Biology, Faculty of Dentistry, University of Oslo, 0372 Oslo, Norway; 3 Department of Biotechnology, Faculty of Applied Ecology, Agricultural Sciences and Biotechnology, Inland Norway University of Applied Sciences, 2317 Hamar, Norway; 4 Department of Virology, Norwegian Institute of Public Health, 0456 Oslo, Norway; 5 Christiania Dental Clinic, Malo Dental, 0188 Oslo, Norway

**Keywords:** S100, EMT, prognosis, biomarkers, proliferation, invasion

## Abstract

S100A16 is a member of the S100 protein family. S100A16 is expressed in a variety of human tissues, although at varying levels. S100A16 expression is especially high in tissues rich in epithelial cells. mRNA and protein levels of S100A16 have been reported to be differentially expressed in the majority of human cancers. Functionally, S100A16 has been linked to several aspects of tumorigenesis, for example, cell proliferation, differentiation, migration, invasion, and epithelial-mesenchymal transition (EMT). Accordingly, S100A16 has been suggested to have both tumour-promoting and suppressive roles in human cancers. S100A16-mediated cellular functions are suggested to be mediated by the regulation of various signaling pathways/proteins including EMT-related proteins E-cadherin and Vimentin, PI3K-AKT, p53, MMP1-1, MMP-2, MMP-9, JNK/p38, etc. In addition to the functional roles, expression of S100A16 has been suggested to have prognostic potential in various cancer types. The aims of this review are to summarise the expression profile, identify common molecular partners and functional roles, and explore the prognostic potential of S100A16 in human cancers.

## 1. Introduction

**Figure 1:**
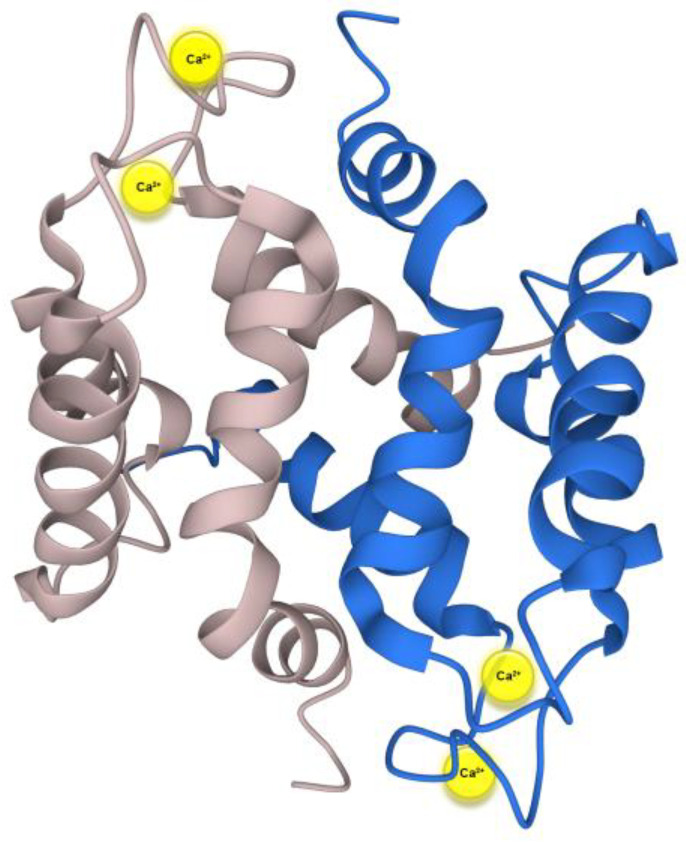
Three-dimensional representation of S100A16 homodimer obtained from the Protein Data Bank in Europe Knowledge Database (PDBe-KB) based on Babini et al. [4,6]. The image has been modified to show the two Ca^2+^ ions (in yellow) that bind to each dimer.

S100A16 is one of the members of the S100 family of calcium-binding proteins. The S100 protein family consists of 25 known members [1]. The *S100A16* gene was originally isolated from an astrocytoma cell line. The S100A16 protein is a small acidic protein comprising 103 amino acids with a molecular weight of 11,801.4 Da and the isoelectric point (p*I*) of 6.28 [2]. This protein is highly conserved in mammals and is ubiquitously expressed in various human tissues [3]. Similar to other S100 proteins, S100A16 contains two EF-hand motifs consisting of a helix-loop-helix structural domain, where the N-terminal domain is interconnected with the C-terminal domain by a “hinge” linker (Figure 1). The two EF-hand motifs are the Ca^2+^ and Zn^2+^ binding sites of S100A6 protein. S100A16 binds to Zn^2+^ with a relatively low affinity at a site different from the Ca^2+^ binding site on the S100A16 protein [4,5]. S100A16 has several unique characteristics compared to other S100 proteins. One of the important features is the presence of only one functional Ca^2+^ binding site in the C-terminal EF-hand, composed of 12 amino acids per protein monomer [2,5]. The N-terminal EF-hand of S100A16 is comprised of 15 amino acids instead of 14, and it lacks the conserved glutamate residue at the last position, a feature possibly related to the dysfunctional Ca^2+^ binding site at the N-terminal region [4,5]. 

Unlike the majority of the other the S100 proteins, S100A16 has been suggested to undergoe only minor conformational changes after calcium binding. S100A16 mostly occurs as a dimer through interaction between helices I, I’, IV, and IV’, forming an X-type helix bundle in both the calcium bound and unbound form. The EF-hand motif does not move to open conformation upon calcium binding. The results of 3D structural analysis suggested that the largest angle changes due to calcium binding occurred between helices II and III which shifted from 157 ± 5° to 144 ± 4°. This shift was very small compared to the other S100 proteins. In addition, the angle shift between helices III and IV upon calcium binding was from 148 ± 3° to 150 ± 4°. The angles between helices I and I’ and helices IV and IV’ is similar on both calcium bound and unbound forms [4]. 

The diverse cellular functions of S100A16 could possibly be explained by interactions with other proteins. Relatively few direct interactions have been identified, largely through high-throughput screening [7]. An investigation into the binding profiles of S100 proteins utilizing a high-throughput holdup assay with synthetic amino acid foldamers revealed a relatively low binding affinity for S100A16 [7]. The most robust interaction so far identified is that of S100A16 and S100A14, which form a heterodimer. This was first identified by utilizing a yeast two-hybrid screen and verified by co-immunoprecipitation [8].

**Figure 2:**
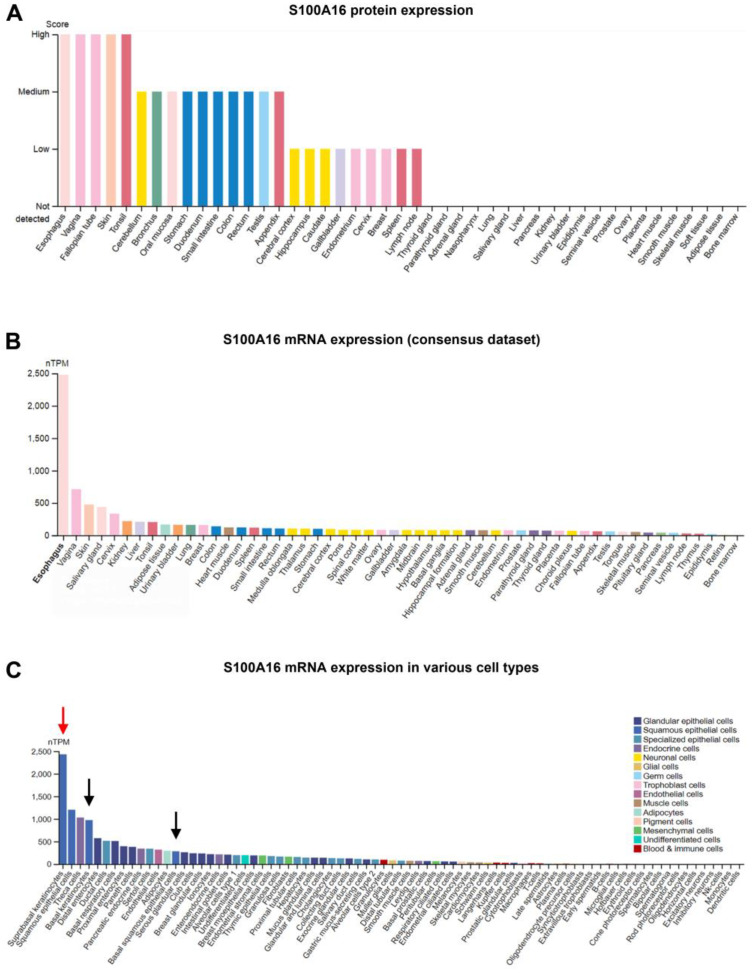
Protein (**A**) and mRNA (**B**) expression data from HPA showed a heterogeneous expression of S100A16 in different normal human tissues. The Consensus mRNA data (**B**), generated from a combination of three datasets (the HPA RNA-seq data, GTEx RNA-seq da RNA-seq data [https://www.gtexportal.org/home/ (accessed on 14 March 2023)], and FANTOM5: the Functional Annotation of Mammalian Genomes 5 data) [14], were obtained from the HPA. (**C**) Normalised S100A16 mRNA expression for different cell type populations showed that suprabasal keratinocytes (red arrow) expressed higher levels of *S100A16* mRNA as compared to basal keratinocytes (black arrows).

Expression of S100A16 has been extensively examined both in normal and malignant tissues [1,2,5,9]. S100A16 has been reported to be expressed in a wide range of normal human tissues, such as the oesophagus, adipose tissue, colon, etc. [2]. At the sub-cellular level, depending on the tissue and cell type, the expression of S100A16 can be membranous, cytoplasmic, nuclear, or a combination [5,8,10]. Similar to the other S100 members, S100A16 has also been suggested to be secreted extracellularly [11]. Analysis of expression data from the Human Protein Atlas (HPA) [12,13] showed high expression of both S100A16 protein and mRNA (HPA consensus dataset) in anatomical sites such as the oesophagus and skin (Figure 2A,B, image available from https://www.proteinatlas.org/ENSG00000188643-S100A16/tissue (accessed on 14 March 2023). 

Similar to S100A14, another S100 protein member, the expression of S100A16 was also found to be enriched in anatomical sites with epithelial lining as compared to tissues with a mesenchymal-stromal phenotype (Figure 2A,B; image available from https://www.proteinatlas.org/ENSG00000188643-S100A16/tissue (accessed on 14 March 2023)). Similar expression patterns can be observed in single-cell transcriptomic data from HPA, where epithelial cells were found to express higher levels of *S100A16* mRNA (Figure 2C, image available from https://www.proteinatlas.org/ENSG00000188643-S100A16/single+cell+type (accessed on 17 March 2023)). Interestingly, within epithelial compartments, the expression of S100A16 was found to be higher in the supra-basal epithelial layers as compared to the basal layer in oral mucosa [11]. In parallel to these observations, the single-cell transcriptomic data from HPA showed higher *S100A16* mRNA expression in suprabasal keratinocytes as compared to basal keratinocytes (Figure 2C, arrow, image available from https://www.proteinatlas.org/ENSG00000188643-S100A16/single+cell+type (accessed on 17 March 2023)). Taken together, the heterogeneous expression pattern of S100A16 with respect to the type and compartment of normal human tissues indicates that S100A16 might have a tissue- and context-specific expression and function.

Compared to normal tissues, the mRNA and protein levels of S100A16 have been reported to be differentially expressed in several cancer types (summarised in Table 1) [2,15]. 

**Figure 3:**
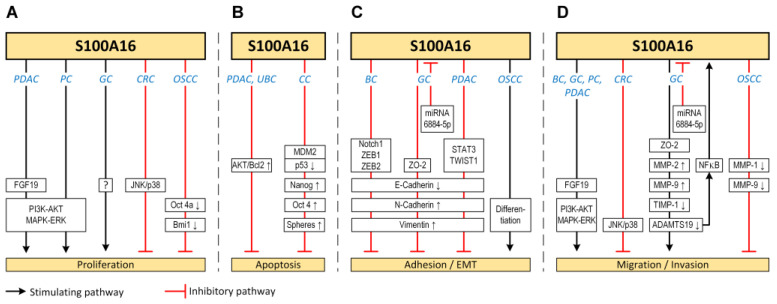
Figure illustrating the functional roles of S100A16 in different cellular processes, such as proliferation (**A**), apoptosis (**B**), adhesion and EMT (**C**), as well as migration and invasion (**D**). Boxes indicate proteins/signaling pathways suggested to be involved in the respective cellular functions. Cancer types are abbreviated in blue text (BC: breast cancer [26,27], CC: cervical cancer [34], CRC: colorectal cancer [20], GC: gastric cancer [18,19], OSCC: oral squamous cell carcinoma [11], PC: prostate cancer [28], PDAC: pancreatic ductal adenocarcinoma [24,35], UBC: urinary bladder cancer [36]).

**Figure 4:**
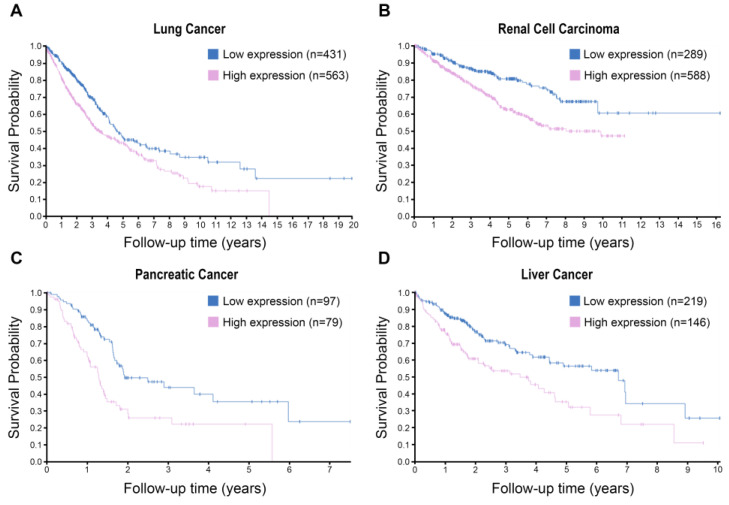
Kaplan-Meier curves (with log-rank test, *p* < 0.001) showing unfavorable survival probabilities for patients with high mRNA expression of *S100A16* in lung cancer (**A**), renal cell carcinoma (**B**), pancreatic cancer (**C**) and liver cancer (**D**). The TCGA mRNA data were used for the analysis, and the images were derived from the Human Protein Atlas.

These observations indicate a functional link between S100A16 and human malignancies. Indeed, several studies have linked S100A16 to the regulation of various cellular functions related to tumorigenesis, as summarised in Figure 3 In addition, S100A16 expression has been suggested to be associated with poor prognosis/survival probabilities in several cancers, as shown in Figure 4 (image available from https://www.proteinatlas.org/ENSG00000188643-S100A16/pathology (accessed on 15 April 2023)). The sections below will provide a comprehensive review of the expression pattern, possible functional roles, and prognostic significance of S100A16 in major human malignancies.

## 2. S100A16 in Human Cancer

### 2.1. Lung Cancer

The expression and functional role of S100A16 are widely studied in different histologic types of lung cancer (LC), predominantly in the non-small cell lung cancer subtypes lung adenocarcinoma (LUAD) and lung squamous cell carcinoma (LUSC). Saito K et al. reported S100A16 as one of the enriched proteins in LUAD cell lines using proteomic analysis [17]. Several studies have reported upregulation of S100A16 mRNA/protein in LC tissues as compared to control/paratumor specimens [15,16,17]. Chen D et al. analyzed DNA CpG methylation sites in *S100A16* in LUAD using TCGA data and reported relative hypomethylation of CpG sites in LUAD lesions compared to the control specimens. These data indicate that the methylation status of *S100A16* might be important for the increased expression of *S100A16* mRNA in LUAD tissues [15].

High expression levels of *S100A16* mRNA have been shown to be associated with poor prognosis in non-small cell lung cancer [15,16,37]. In line with the mRNA results, S100A16 membrane-positive and nucleus-negative expression in LC has been reported to be associated with positive node status, higher T- and tumour stage, poor tumour differentiation, and poor prognosis [37]. In addition, Katono et al. demonstrated that S100A16 immunostaining could be an independent prognostic factor for the overall survival of LUAD patients [38].

Functionally, S100A16 appears to be important in the metastasis of small-cell lung cancer cells to the brain. Xu et al. showed that the expression of S100A16 was upregulated in small cell lung cancer metastases in the brain as compared to the primary tumour lesions, both in humans and in mice [39]. At the molecular level, brain endothelial cell-derived exosomes were found to be responsible for the overexpression of S100A16 in small cell lung cancer cells. S100A16, in turn, promoted the survival of small cell lung cancer cells by preserving mitochondrial integrity and function. Brain endothelial cell-derived EVs were responsible for upregulation of S100A16 in small cell lung cancer, promoting the survival of small cell lung cancer cells [39].

### 2.2. Gastric Cancer

Using publicly available transcriptome datasets such as GEPIA and UALCAN, You and co-authors reported a significantly increased expression of *S100A16* mRNA in gastric cancer (GC) tissue as compared to normal gastric tissue [18]. In line with the above results, the authors found high expression of S100A16 in specimens from GC patients using immunohistochemical analysis [18]. Similar results were also reported by Lv et al. in GC specimens and GC cell lines [19]. 

Functionally, overexpression of S100A16 was reported to increase proliferation and migration of GC cell lines, while opposite effects were reported with knockdown of S100A16. Similar to the in vitro results, S100A16 was found to promote the growth of tumour xenografts in BALB/C nude mice [18]. 

At the molecular level, S100A16 expression was found to be inversely correlated with Zona occludens-2 (ZO-2, a master regulator of cell-to-cell tight junctions) expression in GC specimens and GC cells. Further, S100A16 was found to suppress the expression of ZO-2 through ubiquitylation and degradation, thereby contributing to epithelial-mesenchymal transition (EMT) and the invasion of GC cells [18]. Similar to the above observations, Lv and co-workers have linked overexpression of S100A16 with increased proliferative, invasive, and EMT abilities in GC cells. Further, knockdown of S100A16 was found to be associated with down-regulation of invasion-related proteins such as MMP-2 and MMP-9 as well as up-regulation of TIMP-1. In addition, knockdown of S100A16 was found to suppress the EMT phenotype through upregulation of E-cadherin and concomitant downregulation of N-cadherin and Vimentin. The authors identified miR-6884-5p as an upstream regulator of S100A16 expression in GC cells [19]. Jiang et al. identified ADAMTS19 as an upstream negative regulator of S100A16 expression in GC cancer. Molecularly, ADAMTS19 was found to suppress the phosphorylation and subsequent nuclear translocation of p65, thereby reducing the NF-kB-mediated transcription of S100A16 [40]. 

In addition to having a key functional role, S100A16 has been suggested to be a prognostic marker in GC [18,40]. For example, high expression of S100A16 was reported to be an independent prognostic marker for poor prognosis in GC patients [18]. Similarly, patients with high expression of S100A16 and low expression of ADAMTS19 have been suggested to have poor survival [40].

### 2.3. Colorectal Cancer

Analysing a publicly available transcriptome dataset, Ou and coworkers found significant down-regulation of *S100A16* mRNA in human colorectal cancer (CRC) specimens as compared to normal controls. Similar results were also reported by analysing CRC and corresponding normal controls using immunohistochemistry [20]. Further, studies have reported that low immunoexpression of S100A16 was found to be associated with an aggressive tumour phenotype and poor patient survival, as compared to patients with high expression [21]. In line with the above results, functional studies using CRC cell lines and laboratory mice suggested tumour suppressive functions for S100A16 in CRC [20]. The authors showed that siRNA-mediated silencing of S100A16 increased cell proliferation, migration, and invasion of CRC cells. At the molecular level, the JNK/p38 MAPK signalling pathway was suggested to be involved in S100A16-mediated cellular functions in CRC [20]. 

### 2.4. Pancreatic Cancer

Pancreatic adenocarcinoma, the most common form of pancreatic cancer, is a malignancy with a significantly poor patient survival rate. Several studies have investigated the expression of *S100A16* mRNA and protein levels in pancreatic cancer specimens. *S100A16* mRNA and protein levels were found to be highly expressed in pancreatic adenocarcinoma as compared to normal tissues [22,23,24,25]. Higher expression of S100A16 in pancreatic adenocarcinoma specimens has been reported to be associated with poor patient prognosis [23,24,25,35].

Functionally, S100A16 overexpression has been shown to promote cell proliferation and induce the EMT phenotype and invasive potential of pancreatic adenocarcinoma cell lines both in vitro and in vivo [35]. These functions were suggested to be mediated by S100A16-induced activation of STAT3/TWIST1, AKT, and ERK1/2 signalling pathways [35]. In line with these observations, Fang et al. reported induction of apoptosis and G2/M cell cycle arrest with silencing of S100A16 in a pancreatic adenocarcinoma cell line [24].

Analysis of publicly available transcriptomic datasets has further indicated a possible association between S100A16 and immune infiltrates in the tumour microenvironment. The expression of S100A16 was found to be negatively associated with CD8+T cells [23,25]. These observations indicate that in addition to the direct functional roles in tumour cells, S100A16 might have a role in shaping anti-tumour immunity. 

### 2.5. Breast Cancer

Zhou and co-workers reported upregulation of *S100A16* mRNA and protein levels in breast cancer (BC) specimens and cell lines as compared to the corresponding normal controls [26]. At the sub-cellular level, the S100A16 protein was found to be localised predominantly in the cell membrane in breast cancer cells [27]. Higher expression of S100A16 was reported to be associated with larger tumour sizes, the presence of lymph node metastasis, and poor patient survival. Interestingly, co-expression of S100A16 and S100A14, another member of the S100 protein family, was an independent prognostic factor for poor patient outcome in breast cancer [27]. 

Functionally, overexpression of S100A16 was found to promote proliferation, colony formation, migration, and invasion capabilities of breast cancer cells as compared to control cells [26]. In line with these results, Tanaka et al. reported suppression of migration and invasion of breast cancer cells with knockdown of S100A16, suggesting that S100A16 might have a role in promoting the invasive potential of breast cancer cells [27]. Molecularly, S100A16 was reported to induce the expression of several transcription factors, such as Notch, ZEB1, and ZEB2, involved in the EMT process [26]. The link between S100A16 and EMT was further substantiated by the fact that S100A16 upregulation led to concomitant down-regulation of epithelial markers such as E-Cadherin and beta-Catenin and up-regulation of Vimentin and N-Cadherin [26].

### 2.6. Oral Squamous Cell Carcinoma

Employing a yeast two-hybrid screen, followed by validation with co-immunoprecipitation and reverse co-immunoprecipitation assays, S100A16 was reported to bind with S100A14 in oral squamous cell carcinoma (OSCC) cells. The study found predominantly membranous co-localization of S100A16 and S100A14 in normal oral mucosa and OSCC specimens [9]. Further, over-expression of S100A14 was found to increase the S100A16 protein level but not the mRNA expression levels in the human cancer cell lines studied, indicating a role for S100A14 in possible post-transcriptional regulation of the S100A16 protein. *S100A14* mRNA and protein levels, on the other hand, were found not to be dependent on the expression of S100A16 [9]. 

A strong membranous immunoexpression of S100A16 in the supra-basal (committed/differentiating) epithelial cell layers and negative or weak staining in the basal cell layer (stem cell compartment) in normal oral mucosal tissues suggested a possible link between S100A16 and keratinocyte differentiation [11]. Indeed, the single-cell transcriptomic data from HPA showed higher *S100A16* mRNA expression in suprabasal keratinocytes as compared to basal keratinocytes (Figure 2C). Further, a gradual loss of *S100A16* mRNA and protein levels was found during the progression of OSCC, indicating a possible tumour suppressive function for S100A16 in OSCC. The loss of S100A16 at the tumour-invading front was found to be associated with poor tumour differentiation and reduced patient survival [11]. The association with keratinocyte differentiation was further substantiated by the analysis of microarray datasets, where the mRNA expression of *S100A16* was found to be positively correlated with keratinocyte differentiation markers such as *KRT13*, *IVL*, *TGM1*, and *FLG*. In line with these results, retroviral-mediated overexpression and knockdown of S100A16 in OSCC cells were found to increase and decrease the expression of keratinocyte differentiation markers, respectively. 

In addition to the possible role in differentiation, S100A16 was further found to suppress the cell proliferation, colony formation, and invasion abilities of OSCC cells in vitro. In parallel, expression of S100A16 was found to suppress tumorigenesis of OSCC cells in a mouse xenograft model, and the resulting tumour xenografts demonstrated features of increased differentiation and reduced proliferation/self-renewal. At the molecular level, the tumour suppressive functions of S10016 might be mediated by down-regulation of proliferation/self-renewal markers (Bmi-1 and Oct4A) and MMP-1 and MMP-9 [11].

### 2.7. Prostate Cancer

Both up- and down-regulated expression of S100A16 has been reported in prostate cancer (PC) specimens as compared to the normal control [28,29]. Using real-time PCR and Western blot, Zhu, W et al. reported significant up-regulation of *S100A16* mRNA and protein levels in PC specimens and cell-lines as compared to the control specimens [28]. On the contrary, using the Oncomine database, Wang, R et al. reported down-regulation of *S100A16* mRNA levels in PC specimens [29]. However, the authors found a barely detectable amount of S100A16 in plasma samples of both PC patients and controls. 

Functionally, employing S100A16 over-expression and silencing strategies, S100A16 was suggested to promote proliferation, migration, and invasion of PC cells. These functions were suggested to be mediated by activation of AKT and ERK signalling pathways and down-regulation of cell cycle inhibitors such as p21 and p27 in PC cells [28]. 

### 2.8. Urinary Bladder Cancer

Yao R. and co-workers investigated the mRNA expression of several *S100* gene members in urinary bladder cancer (UBC) specimens from humans, rats, and mice as compared to the respective normal controls. The authors identified *S100A16* mRNA as being overexpressed in UBC specimens from humans, rats, and mice [30]. Using external transcriptome datasets, Guo et al. identified S100A16 as one of the enriched metabolism-associated hub genes in UBC specimens [31]. High *S100A16* mRNA expression levels were suggested to be associated with a poor prognosis in UBC patients. Functionally, down-regulation of S100A16 was reported to suppress migration, invasion, and EMT of UBC cells. At the molecular level, knockdown of S100A16 was shown to reduce the expression of N-cadherin, vimentin, and slug, and increase the expression of E-cadherin in UBC cells [31]. 

Wang et al. investigated the role of S100A16 in chemoresistance in UBC cells. The authors identified S100A16 as a significantly up-regulated protein in a Mitomycin-C-resistant UBC cell line as compared to the parent cell line [36]. Knock-down of S100A16 in the Mitomycin-C-resistant UBC cell line was shown to significantly sensitise the UBC cells to Mitomycin-C. The authors identified Snail as the upstream regulator of S100A16. Furthermore, it was suggested that suppression of Bcl-2 and pAKT/AKT pathways could contribute to the S100A16 knock-down-mediated sensitization of the mitomycin-C-resistant UBC cell line for apoptosis [36]. In addition to the above functional roles, high expression of S100A16 has been reported to be associated with poor patient prognosis. Since chemoresistance is also related to patient prognosis, S100A16 is suggested as a prognostic marker in UBC [36]. 

### 2.9. Renal Cell Carcinoma

Using the TCGA transcriptome dataset, Wang et al. identified *S100A16* mRNA to be significantly up-regulated in renal cell carcinoma (RCC) specimens as compared to noncancerous tissues [32]. High expression of *S100A16* mRNA in RCC specimens was suggested to be associated with poor overall survival, progression-free interval, and disease-specific survival. GO and KEGG analyses further identified VEGF/VEGFR2, PI3K-Akt, and p53/cell cycle as relevant pathways modulated by S100A16. The authors further demonstrated that knockdown of S100A16 suppressed cell proliferation and invasion abilities in RCC cells. These functional effects were suggested to be associated with inhibition of VEGF, VEGFR2, and pAkt in RCC cells with S100A16 knockdown [32]. 

### 2.10. Cervical Cancer

Analysing the TCGA transcriptome dataset using the GEPIA tool, significant up-regulation of *S100A16* mRNA has been reported in cervical cancer (CC) types such as cervical squamous cell carcinoma and endocervical adenocarcinoma specimens as compared to the control specimens [23]. Tomiyama N. and co-workers investigated the role of S100A16 in cancer stem cells using Yumoto cells (a CC cell line). The authors found upregulation of S100A16 in Yumoto cells following sphere formation as compared to monolayer culture. The sphere formation assay is based on the assumption that cancer cells with a high capacity for sphere formation are enriched with cells with high cell-renewal and stemness properties. Using siRNA-mediated silencing of S100A16, the authors suggested that S100A16 was important for the sphere-forming abilities of Yumoto cells and that S100A16 was a positive regulator of stem cell markers such as Oct4 and Nanog [34]. 

In the same line, Zhang et al. reported that S100A16 promoted cell proliferation and invasion properties and the EMT phenotype of the HeLa cell line (CC cell line), probably through the activation of PI3K/Akt signalling pathways [41]. 

### 2.11. Ovarian Cancer

Xu et al., analysing external transcriptome datasets, reported upregulation of *S100A16* mRNA in ovarian cancer (OC) as compared to the corresponding control tissues [33]. High *S100A16* mRNA expression appeared to predict poor patient prognosis in grade II, stage II, and OC patients with *TP53* mutations [33]. Bai Y et al. also reported similar results [42].

## 3. Conclusions

A heterogeneous expression of S100A16 was found in different normal human tissues. Expression of S100A16 was altered in several human malignancies, indicating a possible role for S100A16 in cancer. With the exception of OSCC and CRC, S100A16 was overexpressed in several malignancies, leading to increased proliferation, invasion, and metastasis through a variety of molecular pathways, including PI3K-Akt, MAPK-ERK, JNK/p38, and EMT-signalling. The differential expression pattern of S100A16 and its involvement in key signalling pathways regulating various cellular functions in human cancers suggest that S100A16 might be a promising prognostic marker and a therapeutic target in human cancers.

## Figures and Tables

**Table 1:** Table showing differential expression of S100A16 in different human cancers.

**Cancer Type**	**Specimens**	**mRNA/Protein**	**Expression**	**Ref.**
LC	Specimens *	mRNA and protein	Up	[15]
	Specimens **	mRNA	Up	[16]
	Specimens *	Protein	Up	[17]
GC	GC specimens/cell lines	mRNA and protein	Up	[18,19]
CRC	CRC specimens	mRNA and protein	Down	[20,21]
PC	PC specimens/cell-lines	mRNA and protein	Up	[22,23,24,25]
BC	BC specimens/cell-lines	mRNA and protein	Up	[26,27]
OSCC	OSCC specimens	mRNA and protein	Down	[11]
PC	PC specimens/cell-lines	mRNA and protein	Up	[28]
	PC specimens	mRNA	Down	[29]
UBC	UBC specimens	mRNA	Up	[30,31]
RCC	RCC specimens/cell-lines	mRNA	Up	[32]
CC	CC specimens	mRNA	Up	[23]
OC	OC specimens	mRNA	Up	[33]

* lung adenocarcinoma; ** non-small cell lung cancer; LC: lung cancer; GC: gastric cancer; CRC: colorectal cancer; PC: pancreatic cancer; BC: breast cancer; OSCC: oral squamous cell carcinoma; PC: prostate cancer; UBC: urinary bladder cancer; RCC: renal cell carcinoma; CC: cervical cancer; OC: ovarian cancer.

## Data Availability

Not applicable.

## Abbreviations

ADAMTS19	A disintegrin and metalloproteinase with thrombospondin motifs 19
AKT	RAC-alpha serine/threonine-protein kinase
BC	breast cancer
Bcl2	apoptosis regulator Bcl-2 (B-cell lymphoma 2)
Bmi1	polycomb complex protein BMI-1 (B lymphoma Mo-MLV insertion region 1 homolog)
CC	cervical cancer
CRC	colorectal cancer
E-cadherin	epithelial cadherin
EMT	epithelial-mesenchymal transition
ERK	extracellular signal-regulated kinase
FANTOM	functional annotation of the mammalian genome
FGF19	fibroblast growth factor 19
FLG	filaggrin
GC	gastric cancer
GEPIA	Gene Expression Profiling Interactive Analysis
GO	gene ontology
GTEx	genotype-tissue expression
HPA	human protein atlas
IVL	involucrin
JNK	c-Jun N-terminal kinase
KEGG	Kyoto Encyclopedia of Genes and Genomes
KRT13	keratin 13
LC	lung cancer
LUAD	lung adenocarcinoma
LUSC	lung squamous cell carcinoma
MAPK	mitogen-activated protein kinase
MDM2	E3 ubiquitin-protein ligase Mdm2 (mouse double minute 2 homolog)
miRNA	micro RNA
MMP	matrix metalloproteinase
Nanog	homeobox protein NANOG
N-cadherin	neural cadherin
NFκB	nuclear factor NF-kappa-B
Notch1	neurogenic locus notch homolog protein 1
pAKT	phosphorylated AKT
OC	ovarian cancer
Oct 4a	octamer-binding protein 4
OSCC	oral squamous cell carcinoma
PC	prostate cancer
PDAC	pancreatic ductal adenocarcinoma
PI3K	phosphatidylinositol 3-kinase
p21	cyclin-dependent kinase inhibitor 1
p27	cyclin-dependent kinase inhibitor 1B
p38	p38 mitogen-activated protein kinase
p53	cellular tumour antigen p53
RCC	renal cell carcinoma
Slug	zinc finger protein SNAI2 (protein snail homolog 2/neural crest transcription factor Slug)
Snai1	zinc finger protein SNAI1 (protein snail homolog 1/protein sna)
Stat3	signal transducer and activator of transcription 3
TCGA	The Cancer Genome Atlas
TGM1	protein-glutamine gamma-glutamyltransferase K (transglutaminase-1)
TIMP-1	metalloproteinase inhibitor 1
TP53	tumour protein p53
TWIST1	twist-related protein 1
UBC	urinary bladder cancer
VEGF	vascular endothelial growth factor
VEGFR	vascular endothelial growth factor receptor
ZEB	zinc finger E-box-binding homeobox
ZO-2	tight junction protein ZO-2

## References

[1] Gonzalez L.L., Garrie K., Turner M.D. (2020). Role of S100 proteins in health and disease. Biochim. Biophys Acta Mol. Cell Res..

[2] Marenholz I., Heizmann C.W. (2004). S100A16, a ubiquitously expressed EF-hand protein which is up-regulated in tumors. Biochem. Biophys Res. Commun..

[3] Marenholz I., Heizmann C.W., Fritz G. (2004). S100 proteins in mouse and man: From evolution to function and pathology (including an update of the nomenclature). Biochem. Biophys Res. Commun..

[4] Babini E., Bertini I., Borsi V., Calderone V., Hu X., Luchinat C., Parigi G. (2011). Structural characterization of human S100A16, a low-affinity calcium binder. J. Biol. Inorg. Chem..

[5] Sturchler E., Cox J.A., Durussel I., Weibel M., Heizmann C.W. (2006). S100A16, a novel calcium-binding protein of the EF-hand superfamily. J. Biol. Chem..

[6] Consortium P.-K.B. (2020). PDBe-KB: A community-driven resource for structural and functional annotations. Nucleic Acids Res..

[7] Simon M.A., Bartus É., Mag B., Boros E., Roszjár L., Gógl G., Travé G., Martinek T.A., Nyitray L. (2022). Promiscuity mapping of the S100 protein family using a high-throughput holdup assay. Sci. Rep..

[8] Sapkota D., Costea D.E., Ibrahim S.O., Johannessen A.C., Bruland O. (2013). S100A14 interacts with S100A16 and regulates its expression in human cancer cells. PLoS ONE.

[9] Donato R., Cannon B.R., Sorci G., Riuzzi F., Hsu K., Weber D.J., Geczy C.L. (2013). Functions of S100 proteins. Curr. Mol. Med..

[10] Sapkota D., Bruland O., Parajuli H., Osman T.A., Teh M.T., Johannessen A.C., Costea D.E. (2015). S100A16 promotes differentiation and contributes to a less aggressive tumor phenotype in oral squamous cell carcinoma. BMC Cancer.

[11] Fornander L., Ghafouri B., Kihlström E., Åkerlind B., Schön T., Tagesson C., Lindahl M. (2011). Innate immunity proteins and a new truncated form of SPLUNC1 in nasopharyngeal aspirates from infants with respiratory syncytial virus infection. Proteom. Clin. Appl..

[12] Uhlén M., Björling E., Agaton C., Szigyarto C.A., Amini B., Andersen E., Andersson A.C., Angelidou P., Asplund A., Asplund C. (2005). A human protein atlas for normal and cancer tissues based on antibody proteomics. Mol. Cell. Proteom..

[13] Uhlén M., Fagerberg L., Hallström B.M., Lindskog C., Oksvold P., Mardinoglu A., Sivertsson Å., Kampf C., Sjöstedt E., Asplund A. (2015). Proteomics. Tissue-based map of the human proteome. Science.

[14] Forrest A.R., Kawaji H., Rehli M., Baillie J.K., de Hoon M.J., Haberle V., Lassmann T., Kulakovskiy I.V., Lizio M., Itoh M. (2014). A promoter-level mammalian expression atlas. Nature.

[15] Chen D., Luo L., Liang C. (2018). Aberrant S100A16 expression might be an independent prognostic indicator of unfavorable survival in non-small cell lung adenocarcinoma. PLoS ONE.

[16] Sun L., Zhang Z., Yao Y., Li W.Y., Gu J. (2020). Analysis of expression differences of immune genes in non-small cell lung cancer based on TCGA and ImmPort data sets and the application of a prognostic model. Ann. Transl. Med..

[17] Saito K., Kobayashi M., Nagashio R., Ryuge S., Katono K., Nakashima H., Tsuchiya B., Jiang S.X., Saegusa M., Satoh Y. (2015). S100A16 is a prognostic marker for lung adenocarcinomas. Asian Pac. J. Cancer Prev..

[18] You X., Li M., Cai H., Zhang W., Hong Y., Gao W., Liu Y., Liang X., Wu T., Chen F. (2021). Calcium binding protein S100A16 expedites proliferation, invasion and epithelial-mesenchymal transition process in gastric cancer. Front. Cell Dev. Biol..

[19] Lv H., Hou H., Lei H., Nie C., Chen B., Bie L., Han L., Chen X. (2020). MicroRNA-6884-5p regulates the proliferation, invasion, and EMT of gastric cancer cells by directly targeting S100A16. Oncol. Res..

[20] Ou S., Liao Y., Shi J., Tang J., Ye Y., Wu F., Wang W., Fei J., Xie F., Bai L. (2021). S100A16 suppresses the proliferation, migration and invasion of colorectal cancer cells in part via the JNK/p38 MAPK pathway. Mol. Med. Rep..

[21] Sun X., Wang T., Zhang C., Ning K., Guan Z.-R., Chen S.-X., Hong T.-T., Hua D. (2018). S100A16 is a prognostic marker for colorectal cancer. J. Surg. Oncol..

[22] Zhuang H., Chen X., Dong F., Zhang Z., Zhou Z., Ma Z., Huang S., Chen B., Zhang C., Hou B. (2021). Prognostic values and immune suppression of the S100A family in pancreatic cancer. J. Cell. Mol. Med..

[23] Tu G., Gao W., Li Y., Dian Y., Xue B., Niu L., Yu X., Zhu H. (2021). Expressional and prognostic value of S100A16 in pancreatic cancer via integrated bioinformatics analyses. Front. Cell Dev. Biol..

[24] Fang D., Zhang C., Xu P., Liu Y., Mo X., Sun Q., Abdelatty A., Hu C., Xu H., Zhou G. (2021). S100A16 promotes metastasis and progression of pancreatic cancer through FGF19-mediated AKT and ERK1/2 pathways. Cell Biol. Toxicol..

[25] Chen T., Xia D.M., Qian C., Liu S.R. (2021). Integrated analysis identifies S100A16 as a potential prognostic marker for pancreatic cancer. Am. J. Transl. Res..

[26] Zhou W., Pan H., Xia T., Xue J., Cheng L., Fan P., Zhang Y., Zhu W., Xue Y., Liu X. (2014). Up-regulation of S100A16 expression promotes epithelial-mesenchymal transition via Notch1 pathway in breast cancer. J. Biomed. Sci..

[27] Tanaka M., Ichikawa-Tomikawa N., Shishito N., Nishiura K., Miura T., Hozumi A., Chiba H., Yoshida S., Ohtake T., Sugino T. (2015). Co-expression of S100A14 and S100A16 correlates with a poor prognosis in human breast cancer and promotes cancer cell invasion. BMC Cancer.

[28] Zhu W., Xue Y., Liang C., Zhang R., Zhang Z., Li H., Su D., Liang X., Zhang Y., Huang Q. (2016). S100A16 promotes cell proliferation and metastasis via AKT and ERK cell signaling pathways in human prostate cancer. Tumour Biol..

[29] Wang R., Wu Y., Yu J., Yang G., Yi H., Xu B. (2020). Plasma messenger RNAs identified through bioinformatics analysis are novel, non-invasive prostate cancer biomarkers. Onco Targets Ther..

[30] Yao R., Lopez-Beltran A., Maclennan G.T., Montironi R., Eble J.N., Cheng L. (2007). Expression of S100 protein family members in the pathogenesis of bladder tumors. Anticancer Res..

[31] Guo Y., Zheng Z., Mao S., Yang F., Wang R., Wang H., Liu J., Li C., Wang Q., Zhang W. (2023). Metabolic-associated signature and hub genes associated with immune microenvironment and prognosis in bladder cancer. Mol. Carcinog..

[32] Wang N., Wang R., Tang J., Gao J., Fang Z., Zhang M., Shen X., Lu L., Chen Y. (2022). Calbindin S100A16 promotes renal cell carcinoma progression and angiogenesis via the VEGF/VEGFR2 signaling pathway. Contrast Media Mol. Imaging.

[33] Xu H.Y., Song H.M., Zhou Q. (2020). Comprehensive analysis of the expression and prognosis for S100 in human ovarian cancer: A STROBE study. Medicine.

[34] Tomiyama N., Ikeda R., Nishizawa Y., Masuda S., Tajitsu Y., Takeda Y. (2018). S100A16 up-regulates Oct4 and Nanog expression in cancer stem-like cells of Yumoto human cervical carcinoma cells. Oncol. Lett..

[35] Li T., Ren T., Huang C., Li Y., Yang P., Che G., Luo L., Chen Y., Peng S., Lin Y. (2021). S100A16 induces epithelial-mesenchymal transition in human PDAC cells and is a new therapeutic target for pancreatic cancer treatment that synergizes with gemcitabine. Biochem. Pharmacol..

[36] Wang C., Zhu X., Li A., Yang S., Qiao R., Zhang J. (2019). S100A16 regulated by Snail promotes the chemoresistance of nonmuscle invasive bladder cancer through the AKT/Bcl-2 pathway. Cancer Manag. Res..

[37] Kobayashi M., Nagashio R., Saito K., Aguilar-Bonavides C., Ryuge S., Katono K., Igawa S., Tsuchiya B., Jiang S.-X., Ichinoe M. (2018). Prognostic significance of S100A16 subcellular localization in lung adenocarcinoma. Hum. Pathol..

[38] Katono K., Sato Y., Kobayashi M., Nagashio R., Ryuge S., Igawa S., Ichinoe M., Murakumo Y., Saegusa M., Masuda N. (2017). S100A16, a promising candidate as a prognostic marker for platinum-based adjuvant chemotherapy in resected lung adenocarcinoma. Onco Targets Ther..

[39] Xu Z.H., Miao Z.W., Jiang Q.Z., Gan D.X., Wei X.G., Xue X.Z., Li J.Q., Zheng F., Qin X.X., Fang W.G. (2019). Brain microvascular endothelial cell exosome-mediated S100A16 up-regulation confers small-cell lung cancer cell survival in brain. FASEB J..

[40] Jiang Y., Yu X., Zhao Y., Huang J., Li T., Chen H., Zhou J., Huang Z., Yang Z. (2021). ADAMTS19 suppresses cell migration and invasion by targeting S100A16 via the NF-κB pathway in human gastric cancer. Biomolecules.

[41] Zhang H., Yang Y., Ma X., Xin W., Fan X. (2020). S100A16 regulates HeLa cell through the phosphatidylinositol 3 kinase (PI3K)/AKT signaling pathway. Med. Sci. Monit..

[42] Bai Y., Li L.D., Li J., Lu X. (2018). Prognostic values of S100 family members in ovarian cancer patients. BMC Cancer.

